# Exploiting Adaptive Laboratory Evolution of *Streptomyces clavuligerus* for Antibiotic Discovery and Overproduction

**DOI:** 10.1371/journal.pone.0033727

**Published:** 2012-03-21

**Authors:** Pep Charusanti, Nicole L. Fong, Harish Nagarajan, Alban R. Pereira, Howard J. Li, Elisa A. Abate, Yongxuan Su, William H. Gerwick, Bernhard O. Palsson

**Affiliations:** 1 Department of Bioengineering, University of California San Diego, La Jolla, California, United States of America; 2 Bioinformatics and Systems Biology Graduate Program, University of California San Diego, La Jolla, California, United States of America; 3 Center for Marine Biotechnology and Biomedicine, Scripps Institution of Oceanography and Skaggs School of Pharmacy and Pharmaceutical Sciences, University of California San Diego, La Jolla, California, United States of America; 4 Department of Chemistry and Biochemistry, University of California San Diego, La Jolla, California, United States of America; University of Ottawa, Canada

## Abstract

Adaptation is normally viewed as the enemy of the antibiotic discovery and development process because adaptation among pathogens to antibiotic exposure leads to resistance. We present a method here that, in contrast, exploits the power of adaptation among antibiotic producers to accelerate the discovery of antibiotics. A competition-based adaptive laboratory evolution scheme is presented whereby an antibiotic-producing microorganism is competed against a target pathogen and serially passed over time until the producer evolves the ability to synthesize a chemical entity that inhibits growth of the pathogen. When multiple *Streptomyces clavuligerus* replicates were adaptively evolved against methicillin-resistant *Staphylococcus aureus* N315 in this manner, a strain emerged that acquired the ability to constitutively produce holomycin. In contrast, no holomycin could be detected from the unevolved wild-type strain. Moreover, genome re-sequencing revealed that the evolved strain had lost pSCL4, a large 1.8 Mbp plasmid, and acquired several single nucleotide polymorphisms in genes that have been shown to affect secondary metabolite biosynthesis. These results demonstrate that competition-based adaptive laboratory evolution can constitute a platform to create mutants that overproduce known antibiotics and possibly to discover new compounds as well.

## Introduction

The past ten to fifteen years have seen an alarming increase in the number of infections caused by bacteria such as *Staphylococcus aureus*, *Acinetobacter baumannii*, *Klebsiella pneumoniae*, *Pseudomonas aeruginosa*, and *Mycobacterium tuberculosis* that have evolved resistance to at least one antibiotic [1]. The majority of antibiotics that have been used to treat infections caused by these pathogens are natural products or their semi-synthetic derivatives that originated from bacteria of the order Actinomycetales, in particular the genus *Streptomyces* [2], [3], which are distributed worldwide and are commonly found in soil and marine sediments. This environmental habitat and their ability to produce numerous secondary metabolites have raised many questions about the ecological role of antibiotic production [4], [5], [6], but one prevailing hypothesis is that this ability arose as a product of evolution to serve as a defensive mechanism against microbial competitors [5], [7]. Many new antibiotics were isolated from actinomycetes between the late 1940s and the late 1960s, a period which came to be known as the Golden Age of antibiotic discovery, but this rate plummeted thereafter due in large part to the frequent rediscovery of existing compounds that are highly abundant. Recent genome sequence information, however, suggests that this source is still not yet exhausted. Whole-genome sequencing of several actinomycetes [8], [9], [10], [11], [12] has revealed that each member can synthesize on average 20–30 bioactive small molecules, but only a small fraction of these molecules have ever been detected under various culture conditions. Cloning and heterologous expression of biosynthetic gene clusters [13], [14], interfering with regulatory pathways [15], [16], [17], [18], [19], varying culture conditions [20], [21], co-culturing two or more organisms together [22], [23], [24], [25], [26], [27], [28], and other strategies [29] have all been employed in attempts to stimulate the production of new compounds.

In the laboratory, the genetic and molecular mechanisms underlying a pathogen's ability to develop resistance are frequently studied by serially passing the pathogen over time in media containing increasing concentrations of an antibiotic [30], [31], [32], [33], [34]. This evolution-based strategy leads to mutants with higher levels of resistance when compared to the parental, unevolved clone or to any spontaneous mutants that might have arisen at the outset of the evolution. This general process in which an organism is serially passed against a certain selection pressure to promote adaptation to a new environment is commonly referred to as adaptive (laboratory) evolution, and has been used to generate a number of different phenotypes besides antibiotic resistance. Several examples include the study of *Escherichia coli* as it evolves in glucose minimal media in the laboratory over multiple decades [35], [36], as it adapts to growth on alternative carbon sources such as glycerol and lactate [37], [38], and as it adjusts to the deletion of phosphoglucoisomerase (*pgi*), a major metabolic gene [39], [40]. Adaptive evolution has also found uses as a laboratory tool to investigate mechanisms of ethanol tolerance [41], [42], [43] and osmotic stress [44], to create strains of *Geobacter sulfurreducens* that reduce iron oxide more rapidly [45], to improve yields of commercially-valuable chemicals from metabolically-engineered microbial strains [46], [47], and to examine factors important to virulence and host adaptation among different pathogens [48], [49], [50]. We emphasize here the importance of time and serial passage to this process: these new phenotypes arose only after adaptive laboratory evolution and not after initial, short-term exposure to the selection pressure.

At its core, the current state of antibiotic therapy is one in which a group of pathogenic microorganisms have developed resistance to compounds produced by and isolated from a different group of microorganisms. At the same time, adaptive laboratory evolution is an established technique to generate new phenotypes. Against this backdrop, we hypothesized that microorganisms with extensive secondary metabolism such as actinomycetes could adaptively evolve in the laboratory to produce new antibacterial compounds if they had to compete over time against a drug-resistant pathogen, compounds not normally produced by the wild-type. The use of adaptive laboratory evolution distinguishes our work from prior studies that utilized co-cultures to stimulate production of new compounds [22], [23], [24], [25], [26], [27], [28] but did not utilize repeated serial passage of one organism against another, as was done here. We tested our hypothesis by adaptively evolving multiple colonies of *Streptomyces clavuligerus* against methicillin-resistant *Staphylococcus aureus* (MRSA) N315 [51] over several months in the laboratory. We isolated several evolved *S. clavuligerus* strains during this time, identified the bioactive compound produced by one of them, and resequenced the same strain to detect the genomic mutations that arose during the adaptive evolutionary process.

## Results

### Overview of the method

**Figure 1:**
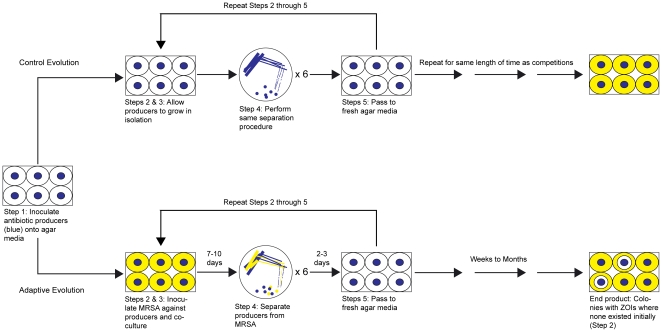
The five-step competition-based adaptive evolution scheme developed here to generate antimicrobial compounds. Antibiotic producing microorganisms are depicted in blue and competing pathogens in yellow. An evolution would end when a larger zone of inhibition (ZOI) appears around an evolved replicate of the antibiotic producer than any ZOI that might surround the unevolved wild-type. As a control, an additional set of replicates are evolved according to the same scheme but are not exposed to the pathogen until the end.

The adaptive laboratory evolution protocol developed here (**Figure 1**) comprises a total of five steps, four of which are performed repeatedly. An inoculum of an antibiotic producer is first deposited onto an agar plate and allowed to grow for several days (Step 1). An inoculum of the target pathogen is then spread onto the plate such that it covers the remaining exposed agar surface and, crucially, completely surrounds and contacts the antibiotic producer (Step 2). Any zone of inhibition (ZOI) surrounding the antibiotic producer initially should be negligible. The plate is then incubated for another several days in order to culture the two bacteria together (Step 3). It is presumed that, over time, competitive exclusion will stimulate the antibiotic producer to synthesize a chemical entity that will inhibit growth of the competing pathogen. After incubation, each replicate of the evolving antibiotic producer is separated from the target pathogen (Step 4). Single colonies are then selected and transferred to a new agar plate (Step 5) to start a new cycle. The latter four steps are performed repeatedly until colonies of the antibiotic producer are isolated that produce a stable ZOI that is larger than any surrounding the original parental clone. During each cycle, the inoculum containing the target pathogen that is used to challenge the antibiotic producer (Step 2) comes from fresh cultures that have had no prior contact with the producer. This requirement ensures that the antibiotic producer faces the same selection pressure during each cycle and that only the producer, not the pathogen, undergoes serial passage and consequent adaptive evolution. The design of this procedure is similar to one used to select for certain *Dictyostelium discoideum* mutants from a mixed population of the same organism [52], but the procedure presented here differs in three critical aspects: 1) two different organisms, rather than two subpopulations of the same organism, are made to compete against each other, 2) one of the two organisms has a proven ability to produce small molecule inhibitors against the other, and 3) the subsequent use of analytical and organic chemistry tools to elucidate the structures of the bioactive molecules.

### S. clavuligerus can evolve along different evolutionary trajectories when competed against MRSA N315 over time

**Figure 2:**
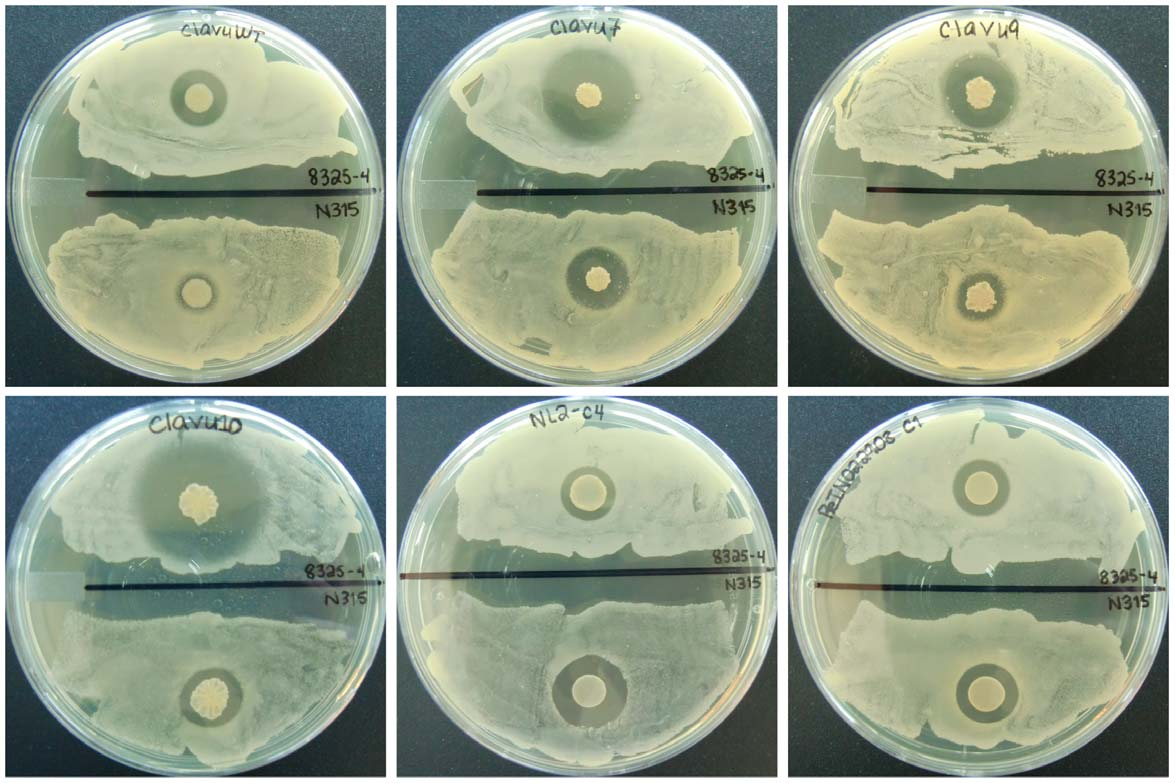
Images of unevolved, wild-type *S. clavuligerus* and five strains (clavu7, clavu9, clavu10, NL2-c4, and ReIN) adaptively evolved against MRSA N315. The top half of each panel shows each strain plated against drug-sensitive *S. aureus* 8325-4 while the bottom half shows the same strain plated against MRSA N315. Top left: wild-type *S. clavuligerus*. Top middle: clavu7. Top right: clavu9. Bottom left: clavu10. Bottom middle: NL2-c4. Bottom right: ReIN. The images were taken 24 hrs after *S. aureus* 8325-4 and MRSA N315 were plated against the six strains.

We tested this adaptive laboratory evolution protocol by competing and adaptively evolving twenty-eight *Streptomyces clavuligerus* replicates against MRSA strain N315 [51] over 4 months, collecting fourteen isolates during this period that appeared to produce a larger ZOI when compared to the small zone surrounding the parental, wild-type strain. These fourteen strains have been designated clavu1 through clavu12, NL2-c4, and ReIN. The first isolates, clavu1, clavu2, NL2-c4, and ReIN, were collected after six weeks and three serial passages. The last isolates, clavu10, clavu11, and clavu12, were collected after four months and eight serial passages. The largest, most stable ZOIs were produced by clavu7, clavu9, clavu10, NL2-c4, and ReIN; thus, we focused our subsequent chemical analysis efforts on these strains. Images of wild-type *S. clavuligerus* and these five evolved isolates co-cultured with *S. aureus* 8325-4, a drug-sensitive research strain, and MRSA N315 are shown in **Figure 2**.

To control against effects arising solely from long-term growth on agar and not from exposure to MRSA N315, we also evolved fourteen additional replicates according to this same procedure but did not expose them to MRSA N315 at any point during their evolution. After three months, we tested the replicates to investigate whether any of them had developed the ability to produce a larger ZOI against MRSA N315, despite the absence of prior contact with the pathogen, than the small, <1 mm ZOI seen surrounding the unevolved clone (**Figure 2**). We defined the size of a ZOI here as the distance from the edge of a *S. clavuligerus* colony to the nearest MRSA N315 colony. One control replicate produced a slightly larger ZOI (1–2 mm) but the remaining thirteen did not; the sizes of the clearing zone surrounding these latter thirteen strains remained unchanged or were smaller when compared to the ZOI surrounding the unevolved clone (**Figure S1**). In contrast, the ZOIs surrounding the fourteen strains evolved against MRSA N315 ranged from two to six millimeters. Several of these strains are shown in **Figure 2**. These data suggest that the ZOIs surrounding the fourteen strains evolved against MRSA N315 arose from exposure to the pathogen and not from long-term growth on the TSA plates (*p* = 0.0071; two-tailed Fisher's Exact Test).

### The antibiotic holomycin can be detected in the ZOI surrounding clavu7 but not in the ZOI surrounding parental, wild-type S. clavuligerus, or four of the other evolved strains

**Figure 3:**
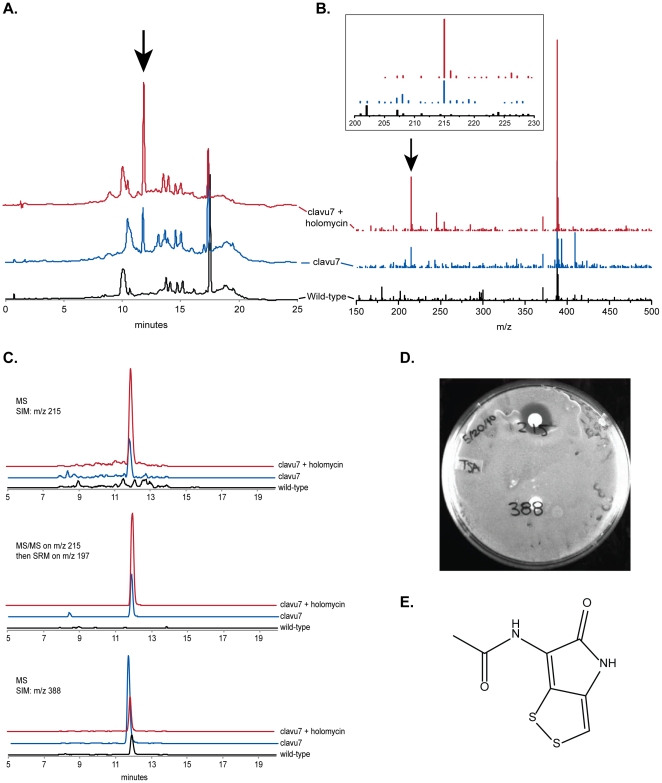
HPLC-MS data from wild-type *S. clavuligerus* (black), clavu7 (blue), and clavu7 spiked with holomycin (red). **A.** HPLC chromatograms of extracts from the three samples. The arrow indicates the peak corresponding to holomycin, which elutes at approximately 11.8 min. Note that this peak is not detected in extracts from wild-type *S. clavuligerus*. **B.** MS total ion monitoring at 11.8 min. There is an intense peak at *m/z* 388 in both wild-type *S. clavuligerus* and clavu7 samples, but an ion with *m/z* 215 ([M+H]^+^), which is holomycin and indicated by the arrow, can be detected in the clavu7 sample only. *Inset*. Magnification of the region surrounding *m/z* 215. **C.** MS and MS/MS select ion monitoring for *m/z* 215 (MS), *m/z* 197 (MS/MS; SRM) and *m/z* 388 (MS). The *m/z* 197 fragment in the MS/MS in particular is a sensitive signature for holomycin. **D.** When holomycin and the *m/z* 388 compound are separated (Figure S1) and tested individually against MRSA N315, only holomycin shows bioactivity. **E.** The structure of holomycin. High-resolution MS/MS indicates that the *m/z* 197 fragment corresponds to loss of H_2_O ([M−H_2_O+H]^+^, Figure S3). Abbreviations: SIM, select ion monitoring; SRM, selected reaction monitoring.

We next focused on one isolate, clavu7, and performed a detailed chemical analysis to establish the identities of the antibacterial compounds that were present in its ZOI. The methanol extracts from clavu7 ZOIs semi-purified by solid-phase extraction (SPE) were loaded onto an HPLC column and fractions collected in half-minute intervals over the entire course of the separation. Only one of these fractions, eluting at 11.8 minutes, showed bioactivity against MRSA N315 and corresponded to a single peak in the chromatogram (**Figure 3**). Moreover, this peak was not present when identical-sized agar zones surrounding wild-type *S. clavuligerus* colonies were extracted in the same manner (**Figure 3**). Comparative mass spectrometric (MS) analysis revealed that an ion with *m/z* 388 eluted from both samples at this time point but that the clavu7 sample had an additional ion with *m/z* 215 ([M+H]^+^) (**Figure 3**).

We isolated the compound with *m/z* 215 from the compound with *m/z* 388 to establish definitively whether it had antibacterial activity (**Figure S2**). The isolated *m/z* 215 compound displayed clear bioactivity against MRSA N315 whereas the *m/z* 388 compound did not (**Figure 3**). We then sought to determine the structure of this bioactive compound by high-resolution MS and MS/MS (**Figure S3**). The molecular ion was found to be C_7_H_7_N_2_O_2_S_2_ ([M+H]^+^; exact mass: 214.9941), which is identical to that of holomycin, an antibiotic *S. clavuligerus* is known to produce [53]. Moreover, the fragment at *m/z* 173 in the high-resolution MS/MS (**Figure S3**), having the molecular formula C_7_H_7_N_2_O_2_S_2_ (exact mass: 172.9836), matched the fragmentation pattern reported previously for holomycin and corresponds to the core dithiolopyrrolone holothin structure [54], [55]. The identity of the compound was subsequently confirmed by comparing the HPLC-MS characteristics of our unknown with that of pure holomycin: both had the same elution time and same MS and MS/MS fragmentation patterns (**Figure 3**).

Clavu7 produces holomycin constitutively. Even when clavu7 colonies were not challenged by MRSA N315, we could still detect holomycin in extracts of the agar zones immediately surrounding clavu7 colonies. The yield from a set of 30 clavu7 plates was routinely 3–4 nmol, but this amount was affected by batch-to-batch variability in the growth medium.

Having established a definitive protocol to isolate and identify holomycin, we next examined whether clavu9, clavu10, NL2-c4, and ReIN produce this compound as well. When their surrounding ZOIs were extracted and analyzed via LC-MS using the same procedure developed for clavu7, we could detect low amounts of holomycin from NL2-c4. When this fraction was collected off the HPLC and tested against MRSA N315, however, it was not bioactive. No holomycin could be detected during LC-MS analysis of extracts from the other strains. We therefore conclude that clavu9, clavu10, NL2-c4, and ReIN produce a different suite of bioactive compounds than clavu7 does.

### At least six mutations distinguish the genome of clavu7 from the reference genome: the loss of a 1.8 megabase (Mbp) plasmid and five single nucleotide polymorphisms (SNPs)

**Figure 4:**
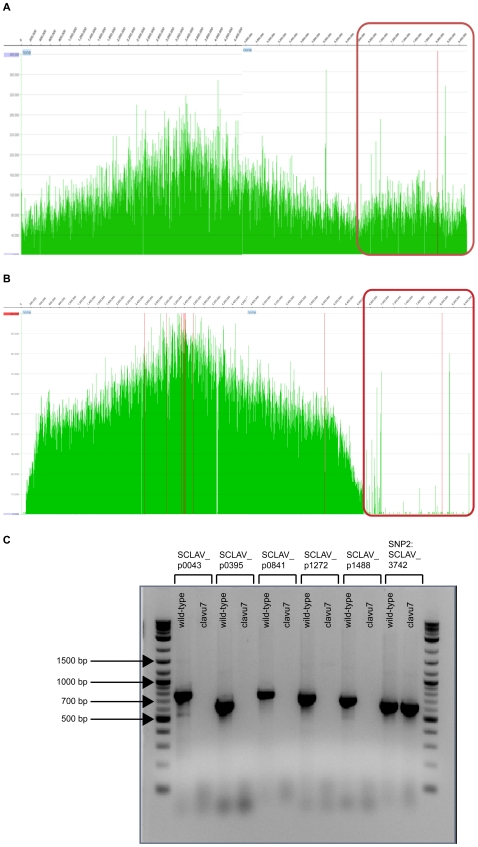
Alignment of wild-type *S. clavuligerus* and clavu7 resequencing data to the reference *S. clavuligerus* ATCC 27064 genome. **A.** Alignment of Illumina sequencing data from the wild-type *S. clavuligerus* strain used in this study to initiate adaptive evolution against MRSA N315. As expected, the data maps to the entire reference sequence. The encircled region indicates the position of the 1.8 Mbp pSCL4 plasmid. **B.** Alignment of Illumina data from clavu7. There is negligible read coverage for pSCL4. **C.** PCR amplification of five pSCL4 amplicons and one chromosomal amplicon from both wild-type *S. clavuligerus* and clavu7. The five pSCL4 amplicons can be amplified using DNA from wild-type *S. clavuligerus* as a template but not from clavu7 DNA, results which reinforce the Illumina sequencing data. The chromosomal amplicon serves as a positive control and corresponds to the same amplicon used to Sanger sequence the SNP in SSCG_05972 (Table 1).

Since biosynthetic gene clusters encode antibiotic production, we sequenced clavu7 and the parental, wild-type *S. clavuligerus* strain to identify any mutations that might have arisen during adaptation. We detected six mutations in clavu7 (**Table 1**). Of note, we confirmed that the wild-type *S. clavuligerus* strain we used to start the evolutions (ATCC 27064) contained a 1.8 Mbp linear plasmid designated pSCL4 [10] by mapping Illumina sequence data collected for the wild-type strain onto the published reference sequence (**Figure 4A**). On the other hand, there was negligible read coverage of the megaplasmid when Illumina data from clavu7 was mapped onto the same reference sequence (**Figure 4B**). Furthermore, we could successfully PCR amplify five amplicons located on pSCL4 from genomic DNA extracted from wild-type *S. clavuligerus* but not from clavu7 (**Figure 4C**). Both of these data sets support the concept that clavu7 has lost pSCL4. The size of the wild-type *S. clavuligerus* chromosome is approximately 6.8 Mbp [10], [12]; therefore, the loss of pSCL4 reduced the total clavu7 genome size by 21%.

**Table 1:** List of mutations detected in clavu7.

	Broad locus ID	DSM locus ID	Annotation	SNP
1			Loss of pSCL4 megaplasmid	
2	SSCG_02612	SCLAV_2674	WD-40 repeat-containing protein	C1096T
3	SSCG_05972	SCLAV_3742	malate dehydrogenase	C570A
4	SSCG_00146	SCLAV_4200	N-(5-amino-5-carboxypentanoyl)-L-cysteinyl-D-valine synthase	G4573C
5	SSCG_05988	SCLAV_5196	glycosyl hydrolase	G1245A
6	SSCG_06722	SCLAV_5104	pyrroloquinoline quinone biosynthesis protein B	G433A

The Broad locus IDs are taken from accession number ABJH00000000. The DSM locus IDs are taken from accession number ADGD00000000. The SNP positions are based on Broad locus IDs.

This observation prompted us to investigate whether pSCL4 had disappeared from any of the other strains evolved against MRSA N315 or from the fourteen control replicates evolved for the same amount of time. A possible link between the loss of an unidentified extrachromosomal element, which we hypothesized might have been pSCL4, and a holomycin-producing *S. clavuligerus* mutant motivated this investigation [56]. We attempted to detect the megaplasmid by PCR amplification using the same five primer sets that were used to verify the loss of pSCL4 from clavu7 (**Table S1**). We could not detect pSCL4 in clavu9, and we could detect only the rightmost portion of it in clavu10 (**Figure S4**). In contrast, we could detect all five amplicons in NL2-c4 and ReIN, and in all fourteen control replicates (**Figure S4**). These data do not support a link between the loss of pSCL4 and holomycin overproduction. On the other hand, the data suggest that exposure to MRSA N315 might have promoted the loss of pSCL4 from clavu7 and clavu9 since all fourteen control replicates still retain the megaplasmid (*p* = 0.039; two-tailed Fisher's Exact Test).

In addition to loss of pSCL4, a second notable mutation is the nonsynonymous SNP in ACV synthetase (*pcbAB*), raising the possibility that production of β-lactam antibiotics might be perturbed in clavu7. This enzyme is a three-module nonribosomal peptide synthetase (NRPS) that catalyzes the second step in β-lactam biosynthesis in actinomycetes, the condensation of α-aminoadipic acid, l-cysteine, and l-valine to form the tripeptide l-*δ*-*α*-AAA-l-cysteinyl-d-valine (LLD-ACV) [57]. The pathways downstream of LLD-ACV eventually lead to the biosynthesis of several major β-lactams such as penicillin G, cephalosporin C, and cephamycin C. Bioinformatics analysis of the *S. clavuligerus* ACV synthetase suggests that the SNP occurs within the second of three adenylation domains (**Figure S5**).

Besides ACV synthetase, there are two SNPs in genes involved in primary metabolism, malate dehydrogenase (*mdh*) and glycosyl hydrolase. The annotations for both suggest that they are synonymous mutations. Another SNP is located in a putative gene containing a WD-40 repeat unit and would result in a proline to serine substitution. The final SNP is a nonsynonymous mutation in pyrroloquinoline quinone biosynthesis protein B (*pqqB*).

The holomycin biosynthetic gene cluster in *S. clavuligerus* has recently been identified [58], but we could not detect any mutations within this cluster in clavu7.

## Discussion

Adaptation is normally seen as the enemy of the antibiotic discovery and development process in part because adaptation among pathogens leads to resistance. In contrast, the method presented here exploits the power of adaptation among antibiotic producers to accelerate the discovery of antimicrobial compounds. Accordingly, there are three major findings of this study. The first is the development of a novel competition-based adaptive laboratory evolution method to elicit the production of antibacterial compounds. The use of adaptive evolution distinguishes this method from prior studies aimed at stimulating secondary metabolite production through the co-culture of two or more organisms [22], [23], [24], [25], [26], [27], [28]. In such studies, two or more organisms are normally cultured together and the supernatant assayed for the presence of bioactive compounds not normally produced when the organisms are cultured separately. If no bioactive compound is detected from the mixed culture, the supernatant is typically discarded. In the method developed here, however, the absence of bioactivity after the initial co-culture does not signal the end of the experimental process. Instead, we isolate the microorganism of interest from the mixed culture and then compete it against the second (target) microorganism a second time. This cycle of co-culture and isolation repeats itself over multiple rounds until bioactivity can be detected, and is a laboratory-based implementation of the competitive exclusion principle [59], [60], [61]. The second is a proof-of-principle demonstration of the method, exhibited through the isolation of a set of evolved *S. clavuligerus* mutants that produce larger ZOIs against MRSA N315 than the parental wild-type clone does, one of which produces bioactive amounts of holomycin. The third is a demonstration through the same mutant that a very large plasmid constituting 21% of the genome content of *S. clavuligerus*, pSCL4, can be successfully removed from the wild-type organism. This megaplasmid contains 25 clusters that encode putative secondary metabolites [10] but, interestingly, clavu7 evolved against MRSA N315 by eliminating the plasmid rather than utilizing any of the 25 possible products encoded by the plasmid-borne clusters.

Holomycin is a member of the pyrrothine class of antibiotics and has been shown to inhibit growth of several drug-sensitive and drug-resistant strains of *S. aureus* [62]. Although the precise regulatory programming that controls holomycin biosynthesis in *S. clavuligerus* remains to be elucidated, certain defined mutants in which genes catalyzing the late steps in clavulanic acid biosynthesis have been deleted have been shown to overproduce holomycin [54]. The data presented here, however, point to several other factors that might boost holomycin production. The SNP in *pqqB*, pyrroloquinoline quinone biosynthesis protein B, is particularly noteworthy because PqqB is involved in the biosynthesis of pyrroloquinoline quinone [63], a redox cofactor for several bacterial dehydrogenases such as glucose dehydrogenase, methanol dehydrogenase, and possibly several other alcohol dehydrogenases [63], [64], [65]. Intriguingly, a *pqqB* deletion mutant of *Pseudomonas fluorescens* overproduces the antibiotic/antifungal compound pyoluteorin [66], suggesting that a connection exists between the *pqq* operon and secondary metabolite production in bacteria. Based on these data, we speculate the following mechanism to explain the increased holomycin production seen in clavu7. First, the SNP in *pqqB* perturbs the biosynthesis of pyrroloquinoline quinone, which in turn perturbs the enzymatic activity of multiple dehydrogenases, including possibly malate dehydrogenase since clavu7 contains a SNP in this enzyme as well. This mutant form of malate dehydrogenase is then able to synthesize greater amounts of oxaloacetate, the TCA cycle precursor to aspartate and by extension to cysteine. Because each molecule of holomycin is formed from two molecules of cysteine [58], higher cysteine levels would allow clavu7 to biosynthesize larger quantities of the antibiotic. Additional data would be needed to confirm this proposed mechanism.

The appearance of other SNPs in clavu7 suggests that other aspects of primary and secondary metabolism are also perturbed in this strain. According to current genome annotations for *S. clavuligerus* (GenBank accession numbers ADGD00000000 and ABJH00000000), the SNP in ACV synthetase would result in a valine to leucine substitution within the adenylation domain of the second module to the N-terminal side of a conserved amino acid binding pocket (**Figure S5**). Consequently, the substitution does not appear to affect any of the eight or ten critical residues within the binding pocket that mediate amino acid specificity [67], [68], suggesting that cysteine continues to be incorporated into the second module of the mutant ACV synthetase. Given this assessment, this SNP might be a neutral mutation; however, data from other adaptive evolution experiments, most involving the model bacterium *Escherichia coli*, argue against this viewpoint. For example, when all combinations of mutations that were detected after several adaptive evolution studies were introduced back into the parental strain, every mutation was found to impact the endpoint phenotype either singly or in combination [37], [38], [39]. For this reason, we posit that the SNP in ACV synthetase does not change the substrate specificity of the module but that it might affect another property of the enzyme such as reaction kinetics.

Proteins containing WD-40 motifs are common scaffolding proteins in eukaryotes that mediate protein-protein interactions [69], [70]. These proteins also appear frequently in actinobacteria (especially actinomycetes), cyanobacteria, and proteobacteria; however, they are rare among other prokaryotes, so the appearance of a SNP in a WD-40 containing protein in clavu7 (**Table 1**: SCLAV_2674) is notable. A WD motif consists of approximately 40 amino acids that often terminates in a tryptophan-aspartate (W-D) dipeptide, and there is another characteristic dipeptide sequence (VH, AH, SH, or GH) located approximately 27 base pairs to the N-terminal side of the WD marker [71]. The ∼27 amino acids between the two markers form a conserved core region [71]. A single WD-40 containing protein normally has 4–16 WD-40 units, and there appears to be three of them in SCLAV_2674 (**Figure S6**). The SNP in SCLAV_2674 in clavu7 results in a P366S amino acid substitution that is located to the N-terminal side of the three repeats. In eukaryotes, proteins containing WD-40 motifs are known to play a role in diverse processes such as signaling, cell cycle control, and cytoskeleton assembly [69], [70], and we speculate that they might play a role in the assembly of multi-protein complexes involved in secondary metabolite biosynthesis as well.

Prior to elimination, several regions from pSCL4 might have been transferred to the chromosome since some reads from the Illumina data for clavu7 still mapped to the plasmid sequence (**Figure 4B**). This observation does not appear to be an artifact of library preparation or other aspects of the sample handling and sequencing process; two different colonies sequenced one year apart using two different sample preparation protocols and reagents yielded the same result.

In conclusion, we develop and demonstrate a competition-based adaptive laboratory evolution method here that pitted *S. clavuligerus* against MRSA N315 and show that this method can lead to the biosynthesis of a known antibiotic, holomycin, that is not produced in detectable quantities by the wild-type strain when grown under identical conditions. Looking forward, the strengths of this approach could extend beyond this particular example. First, this method is a general, systematic platform technique that in theory can be used to overproduce or possibly discover new antibacterial compounds against other pathogens, not just MRSA. Several possibilities include pathogenic fungi [72], potential biowarfare agents such as *Yersinia pestis and Francisella tularensis*, and many drug-resistant Gram-negative bacteria such as *A. baumannii, K. pneumoniae*, and *P. aeruginosa*. The latter group represents a particularly intriguing target choice because their extensive drug resistance profile increases the probability that new chemical entities would be discovered rather than existing compounds. Moreover, this method can be applied to other fields besides medical microbiology since the principal requirement for implementation is simply the ability to culture both the organism of interest and the target on the same growth medium. One possible example is the discovery of agents to control plant pathogens important to agriculture. Second, parallel evolutions descended from the same starting clone might lead to different, alternative phenotypes as replicates traverse multiple paths through the evolutionary landscape. In the case of antibacterial discovery from actinomycetes, this phenomenon could lead to the overproduction or discovery of multiple, different inhibitors for each parental strain that undergoes adaptive evolution. Indeed, preliminary data from clavu9, clavu10, ReIN, and NL2-c4 support this viewpoint since we could not detect bioactive amounts of holomycin from extracts of these four strains, suggesting that they produce at least one other bioactive compound. Third, this approach is amenable to high-throughput implementation through the use of liquid handlers, colony pickers, and other automated equipment to evolve and screen multitudes of colonies. Since many commercially valuable natural products are obtained through fermentation of various actinomycetes, this tactic could provide insights into new mechanisms to increase their yields – and potentially to lead to the discovery of new compounds.

## Materials and Methods

### Bacterial strains

*Streptomyces clavuligerus* 27064 was purchased from ATCC. Methicillin-resistant *Staphylococcus aureus* 8325-4 and N315 were a gift from Victor Nizet (Univ. of California, San Diego). The strains clavu1 through clavu12, NL2-c4, and ReIN were all generated during this study.

### Chemicals and supplies

Trypticase soy broth with dextrose (TSB) [Becton Dickinson, Sparks, MD] and trypticase soy agar (TSA) were used throughout this study (both 30 g/L) and dissolved in deionized water prior to autoclaving. To make TSA, we added 18 g/L Bacteriological Agar (Sigma-Aldrich, St. Louis, MO) to TSB. To extract holomycin, we used ACS-grade methanol (Fisher Scientific, Pittsburgh, PA), ExpressPlus 0.22 µm membranes for sterile filtration (Millipore, Billerica, MA) and Oasis MCX cation exchange solid-phase extraction (SPE) columns (Waters Corp., Waltham, MA). The amount of holomycin we extracted from ZOIs surrounding clavu7 colonies was affected by batch-to-batch variability in the TSB even though we always sourced from the same supplier.

### Adaptive evolution protocol

The general, fundamental aspects of the adaptive evolution protocol developed here is described in the main text and depicted in **Figure 1**. This section will focus on specific details concerning its implementation with respect to *S. clavuligerus* against MRSA N315. The steps noted in this section correspond to those shown in **Figure 1**.

The *S. clavuligerus* vs. MRSA N315 evolutions began by inoculating 50 mL of TSB in a 250 mL triple-baffled flask with wild-type *S. clavuligerus* mycelia that were stored in 20% glycerol at −80°C. The flask was capped with a foam plug, placed on top of a magnetic stir plate and incubated at 30°C. A magnetic stir bar spinning inside the flask was used for aeration.

After visible growth of the pre-culture, an aliquot was diluted such that its optical density at 600 nm (OD600) was 0.10–0.13. Two microliters were then deposited onto each of seven spots spaced equidistantly on a 100 mm×15 mm TSA plate and incubated at 28°C for three days (Step 1). On the third day, we plated approximately 150 µL of MRSA N315 suspended in TSB onto each of the plates (Step 2). The density of the MRSA N315 inocula was 0.01 (range: 0.008–0.013), and care was taken to ensure that the inocula completely surrounded and came into contact with each *S. clavuligerus* colony. At the same time, however, we attempted to minimize the amount of MRSA N315 inocula that flowed over the top of the colonies. The plates were again incubated at 28°C, this time for 5–7 days (Step 3), after which each *S. clavuligerus* colony was streaked onto fresh TSA plates to separate them from MRSA N315 (Step 4). After incubating for another 2–3 days, small fragments from single *S. clavuligerus* colonies belonging to each replicate were broken off and transferred to a new TSA plate such that there were once again seven replicates spaced equidistantly apart per plate (Step 5). After another 3 days incubation, fresh MRSA N315 was again plated against the *S. clavuligerus* colonies to continue the cycle of adaptive evolution. Importantly, we serially passed *S. clavuligerus* only; we did not pass MRSA N315 along with *S. clavuligerus*. In other words, the MRSA N315 that was plated during each cycle came from cultures that had never come into previous contact with any of the *S. clavuligerus* replicates.

In total, we evolved 28 replicates on four TSA plates in this manner over a four-month period and collected 14 isolates that appeared to produce larger ZOIs against MRSA N315 when compared to wild-type *S. clavuligerus*. As a control, fourteen additional replicates were also evolved in this manner, but they were not exposed to MRSA N315 at any point during their evolution. To store each evolved isolate long-term, we re-streaked them on fresh TSA plates, transferred a well-isolated colony into TSB, and incubated the culture with spinning to enable liquid growth. Glycerol was then added to aliquots of the mycelia to a final concentration of 20%. The samples were then frozen and stored at −80°C.

### LC-MS analysis of bioactive components from clavu7 ZOIs and identical-sized zones surrounding wild-type S. clavuligerus colonies

Frozen clavu7 mycelial stocks were regrown by inoculating them into 50 mL TSB in 125 mL triple-baffled flasks with spinning. Once there was visible growth, and as long as the cells were still in exponential phase, the OD600 of the pre-culture was adjusted to 0.1–0.13 before plating on TSA. If the density of the pre-culture corresponded to stationary phase densities, we discarded the pre-culture and made a new one from frozen stock.

To extract compounds from the ZOI surrounding clavu7 colonies, we first deposited 2 µL of the OD600 0.1–0.13 pre-cultures onto each of 16 spots on TSA plates. We routinely used 30 such plates every time we carried out an extraction. The plates were then incubated at 28°C for three days, conditions identical to those used during adaptive evolution. Because we did not know *a priori* whether the bioactive compound produced by clavu7 was induced by the presence of MRSA N315 or whether it was produced constitutively, we initially plated MRSA N315 on every clavu7 plate three days after clavu7 was inoculated. Again, this step is identical to that used during adaptive evolution (Step 2), but was later skipped when performing extractions (see below). After another 24 h incubation, ZOIs surrounding each clavu7 colony were punched out with a cork borer, collected together along with the clavu7 colonies, and diced into small pieces. The mixture comprising macerated agar (ZOIs) and clavu7 colonies was then extracted with methanol for approximately 30 min with gentle spinning on a stir plate. The methanol extract was then sterile-filtered through a 0.22 µm membrane to remove bacteria and rotavapped at 37°C until 2–3 mL remained. Deionized water was subsequently added to the concentrated methanol sample according to the ratio sample∶water 1 mL∶39 mL (2.5% final methanol concentration) in order to load the sample onto Oasis MCX SPE columns. We prepped the SPE column with 4 mL methanol followed by 4 mL deionized water before loading the sample. We then washed the SPE column with 2% formic acid in water and then with 20∶80 methanol∶water before eluting with 3 mL 70∶30 methanol∶water. The flow rate was held at approximately 4 mL/minute. Subsequently, we concentrated the 70∶30 methanol∶water fraction through use of a speed-vac set to 37°C until 50–100 µL remained.

This semi-purified sample was then subjected to LC-MS and LC-MS/MS analysis using an Agilent C-18 column (Eclipse XDB-C18, 5 µm, 4.6 mm×150 mm) attached to a Thermo Finnigan LCQ Deca system with electrospray ionization in the positive ion mode. The flow rate was 1.0 mL/minute. With solvent A as 5% methanol in water and solvent B as 100% methanol, the mobile phase was: hold 5% B for 2 min; linear gradient to 50% B over 13 min; linear gradient to 95% B over 3 min; hold 95% B for 2 min; linear gradient back to 5% B over 2 min; and hold 5% B for 3 min. Both methanol and water were HPLC grade. Under these conditions, holomycin elutes at 11.8 min with detection at 360 nm. For high-resolution MS, we used a Thermo Scientific LTQ Orbitrap XL mass spectrometer with electrospray ionization.

Once we determined the elution time for the bioactive compound (holomycin) in the HPLC chromatogram and its MS and MS/MS signatures, we tested whether clavu7 produced it constitutively or whether its production was induced by the presence of MRSA N315. To answer this question, we carried out the extraction protocol described above but did not plate MRSA N315 against clavu7 on the third day of incubation. All other steps in the extraction protocol were kept the same. We found that the LC-MS data remained unchanged. Once we obtained these data, we no longer plated MRSA N315 against clavu7 during all subsequent extractions.

As a control, frozen stocks of wild-type *S. clavuligerus* mycelia were regrown, plated, extracted, and analyzed via LC-MS and LC-MS/MS according to the same protocol developed for clavu7.

### Bioactivity testing

Fractions were tested for bioactivity by first making a lawn of MRSA N315 on a TSA plate using an OD600 ∼0.01 TSB pre-culture (range: 0.008 to 0.013). Thirty microliters of the fraction to be tested was then pipetted onto a 6 mm paper disk, allowed to dry, and then the disk transferred to the agar plate containing MRSA N315. After 16–20 h incubation at 30°C, the plate was visually inspected for the presence of an inhibition zone surrounding the paper disk.

### Alignment of resequencing reads and SNP identification

The reference assembly for wild-type *S. clavuligerus* ATCC 27064 [10] was obtained from GenBank (accession number: ADGD00000000) and consisted of 279 contigs. These contigs were concatenated to build the reference sequence for alignment. Illumina reads from the resequencing runs of both the wild-type and clavu7 were iteratively aligned to this reference using MosaikAligner (http://bioinformatics.bc.edu/marthlab/Mosaik). The number of allowed mismatches in the alignment was iteratively increased from 0 to 5 with the unaligned reads used as the input for the subsequent iteration. The alignments were processed using a custom script written in-house [37] to obtain the fold coverage at single base-pair resolution. In addition, a particular location was deemed polymorphic if the observed nucleotide count was greater than twice the count of the actual nucleotide in the reference sequence at that position. A coverage cutoff of 10× was employed for SNP identification in clavu7, and all SNPs were verified by Sanger sequencing of both wild-type *S. clavuligerus* and clavu7 DNA using the primer sets found in Table S2.

## Supporting Information

Figure S1 **Images of fourteen evolved** ***S. clavuligerus*** **control replicates, the starting, unevolved,** ***S. clavuligerus*** **clone, and clavu7 plated against MRSA N315.** **A.** The fourteen evolved control replicates plated against MRSA N315. The left panel shows replicates one through seven while the right panel shows replicates eight through fourteen. These replicates were evolved for three months as fourteen separate lineages, but they were not exposed to MRSA N315 until the three month period ended and this photo was taken. One replicate, indicated by the arrow, displays a slightly larger ZOI (1–2 mm) against MRSA N315 than the starting, unevolved clone, but the other thirteen have ZOIs that are similar or smaller in size. **B.** Seven identical colonies of unevolved, wild-type *S. clavuligerus* plated against MRSA N315. The size of the ZOI against MRSA N315 is approximately 0.5–1 mm. **C.** Seven identical colonies of clavu7 plated against MRSA N315. Clavu7 displays a larger ZOI (2–3 mm) against the pathogen than both the fourteen evolved control replicates and the starting, unevolved clone. All colonies seen in each portion of this figure were made by depositing 2 µL of an OD600 ∼0.1 culture onto the indicated spot on the agar plate, allowing the colonies to grow for 3 days, and then co-culturing the colonies with MRSA N315 on the third day. Photos were taken one day later. Additional images of unevolved *S. clavuligerus* and clavu7 can be found in Figure 2 in the main text.
(TIF)

Figure S2 **Separation of the compound with** ***m/z*** **215 (holomycin) from the compound with** ***m/z*** **388.** The fraction eluting at 11.8 min (see Figure 3A) was collected and re-injected into the same HPLC system using an optimized mobile phase (30∶70 methanol∶water) to yield this new chromatogram and MS spectra. The flow rate (1.0 mL/min) and detection wavelength (360 nm) were the same. Under these conditions, select ion monitoring (SIM) for the holomycin fragments *m/z* 215 (MS) and *m/z* 197 (MS/MS; SRM) revealed that holomycin elutes at 4.6 min while the *m/z* 388 ion (MS) elutes at 3.5 minutes. The SIM for *m/z* 388 was reduced by 1/5 for a better fit in the figure. Abbreviation: SRM, selected reaction monitoring.
(TIF)

Figure S3 **A.** High-resolution ESI-MS of holomycin, the bioactive compound isolated from clavu7. [M+H]^+^, observed mass: 214.9941, calculated mass: 214.9943; [M+Na]^+^, observed mass: 236.9761, calculated mass: 236.9763. **B.** High-resolution ESI-MS/MS of holomycin (*m/z* 214.99) and key MS/MS fragments. There are three main peaks in the data: [M+H]^+^, observed mass: 214.9941, calculated mass: 214.9943; [M−H_2_O+H]^+^, observed mass: 196.9835, calculated mass: 196.9838; [M−COCH_3_+H]^+^, observed mass: 172.9836, calculated mass: 172.9838. A fragmentation mechanism leading to the latter ion, holothin, has been proposed [55].
(TIF)

Figure S4 **PCR amplification of five pSCL4 amplicons from additional strains evolved against MRSA N315 and from fourteen evolved control replicates.** **A.** PCR amplification from evolved strains clavu9, clavu10, NL2-c4, and ReIN. Clavu9 does not contain the megaplasmid, and a portion of the 5′ end is missing from clavu10. Both NL2-c4 and ReIN contain the full length megaplasmid. **B.** PCR amplification from fourteen control replicates evolved in the absence of MRSA N315 over the same amount of time. All fourteen contain the megaplasmid.
(TIF)

Figure S5 **A.** Module and domain architecture of *S. clavuligerus* ACV synthetase. There are three modules that activate each of the three amino acids. The first module contains two domains and initiates biosynthesis of the LLD-ACV tripeptide. The second and third modules each contain condensation (C) – adenylation (A) – thiolation (T) domains typically found in many non-ribosomal peptide synthetases. The final two domains in the third module are epimerization (E) and thioesterase (Te) domains. The arrow points to the location of the SNP detected in the clavu7 ACV synthetase. **B.** Alignment of the second adenylation domain of *S. clavuligerus* ACV synthetase with the amino acid binding pocket within the GrsA phenylalanine adenylation domain from *B. brevis* [73]. Based on the alignment, the l-cysteine binding pocket in ACV synthetase is predicted to be between residues 1590 and 1690. The putative V1525L substitution therefore lies within the second adenylation domain but to the N-terminal side of the predicted binding pocket [67], [68].
(TIF)

Figure S6 **Location of the ∼27 bp conserved core unit (underlined) from three putative WD-40 repeats in SSCG_02612.** The proline associated with the C1096T SNP detected in this gene in clavu7 (Table 1) is shown as well (highlighted); the SNP results in a P366S amino acid substitution. The annotation for SSCG_02612 is shorter than that for SCLAV_2674 (the latter contains an additional 182 amino acids at the N-terminus), but both have the same reading frame.
(TIF)

Table S1 **Primer sets used to PCR amplify five amplicons from pSCL4.**
(DOC)

Table S2 **Primer sets used to confirm clavu7 SNPs by Sanger sequencing.**
(DOC)
