# Supplementary figures and images for: Exploiting Adaptive Laboratory Evolution of Streptomyces clavuligerus for Antibiotic Discovery and Overproduction
Source: PLoS One. 2012 Mar 21;7(3):e33727. doi: 10.1371/journal.pone.0033727 (PMC3312335; doi:10.1371/journal.pone.0033727)

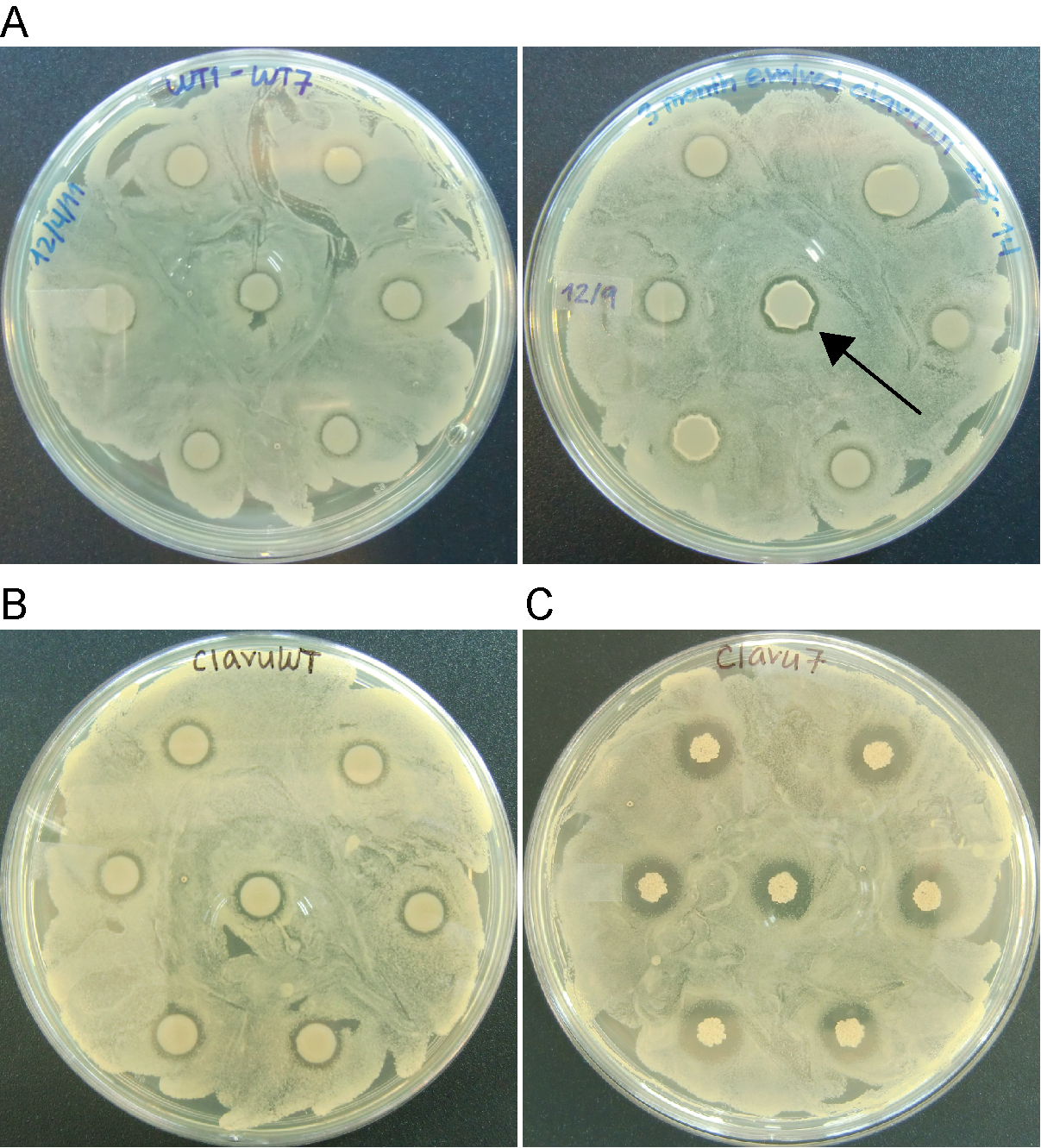

Supplement: Figure S1 — Images of fourteen evolved S. clavuligerus control replicates, the starting, unevolved, S. clavuligerus clone, and clavu7 plated against MRSA N315. A. The fourteen evolved control replicates plated against MRSA N315. The left panel shows replicates one through seven while the right panel shows replicates eight through fourteen. These replicates were evolved for three months as fourteen separate lineages, but they were not exposed to MRSA N315 until the three month period ended and this photo was taken. One replicate, indicated by the arrow, displays a slightly larger ZOI (1–2 mm) against MRSA N315 than the starting, unevolved clone, but the other thirteen have ZOIs that are similar or smaller in size. B. Seven identical colonies of unevolved, wild-type S. clavuligerus plated against MRSA N315. The size of the ZOI against MRSA N315 is approximately 0.5–1 mm. C. Seven identical colonies of clavu7 plated against MRSA N315. Clavu7 displays a larger ZOI (2–3 mm) against the pathogen than both the fourteen evolved control replicates and the starting, unevolved clone. All colonies seen in each portion of this figure were made by depositing 2 µL of an OD600 ∼0.1 culture onto the indicated spot on the agar plate, allowing the colonies to grow for 3 days, and then co-culturing the colonies with MRSA N315 on the third day. Photos were taken one day later. Additional images of unevolved S. clavuligerus and clavu7 can be found in Figure 2 in the main text. (TIF) [file pone.0033727.s001.tif]

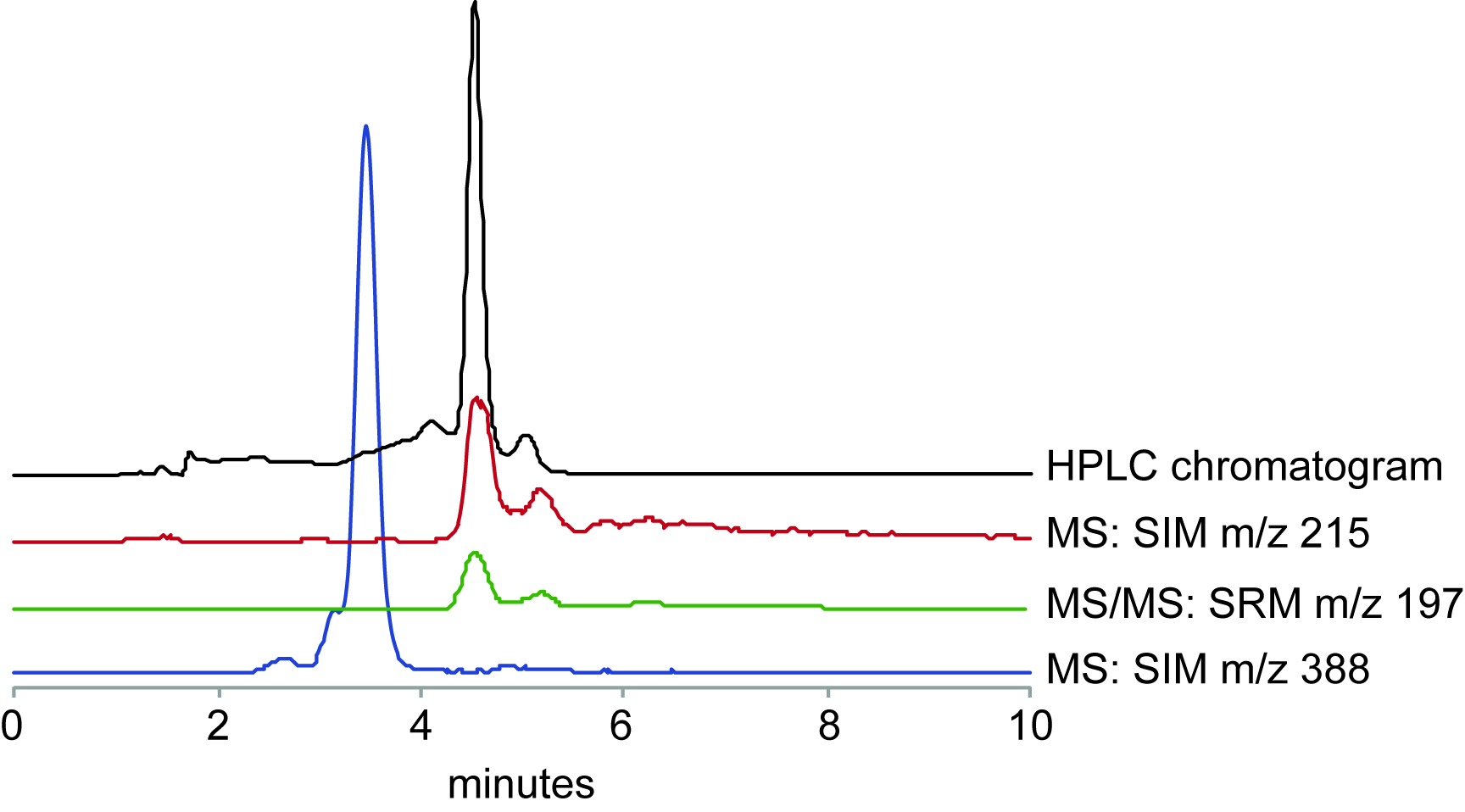

Supplement: Figure S2 — Separation of the compound with m/z 215 (holomycin) from the compound with m/z 388. The fraction eluting at 11.8 min (see Figure 3A) was collected and re-injected into the same HPLC system using an optimized mobile phase (30∶70 methanol∶water) to yield this new chromatogram and MS spectra. The flow rate (1.0 mL/min) and detection wavelength (360 nm) were the same. Under these conditions, select ion monitoring (SIM) for the holomycin fragments m/z 215 (MS) and m/z 197 (MS/MS; SRM) revealed that holomycin elutes at 4.6 min while the m/z 388 ion (MS) elutes at 3.5 minutes. The SIM for m/z 388 was reduced by 1/5 for a better fit in the figure. Abbreviation: SRM, selected reaction monitoring. (TIF) [file pone.0033727.s002.tif]

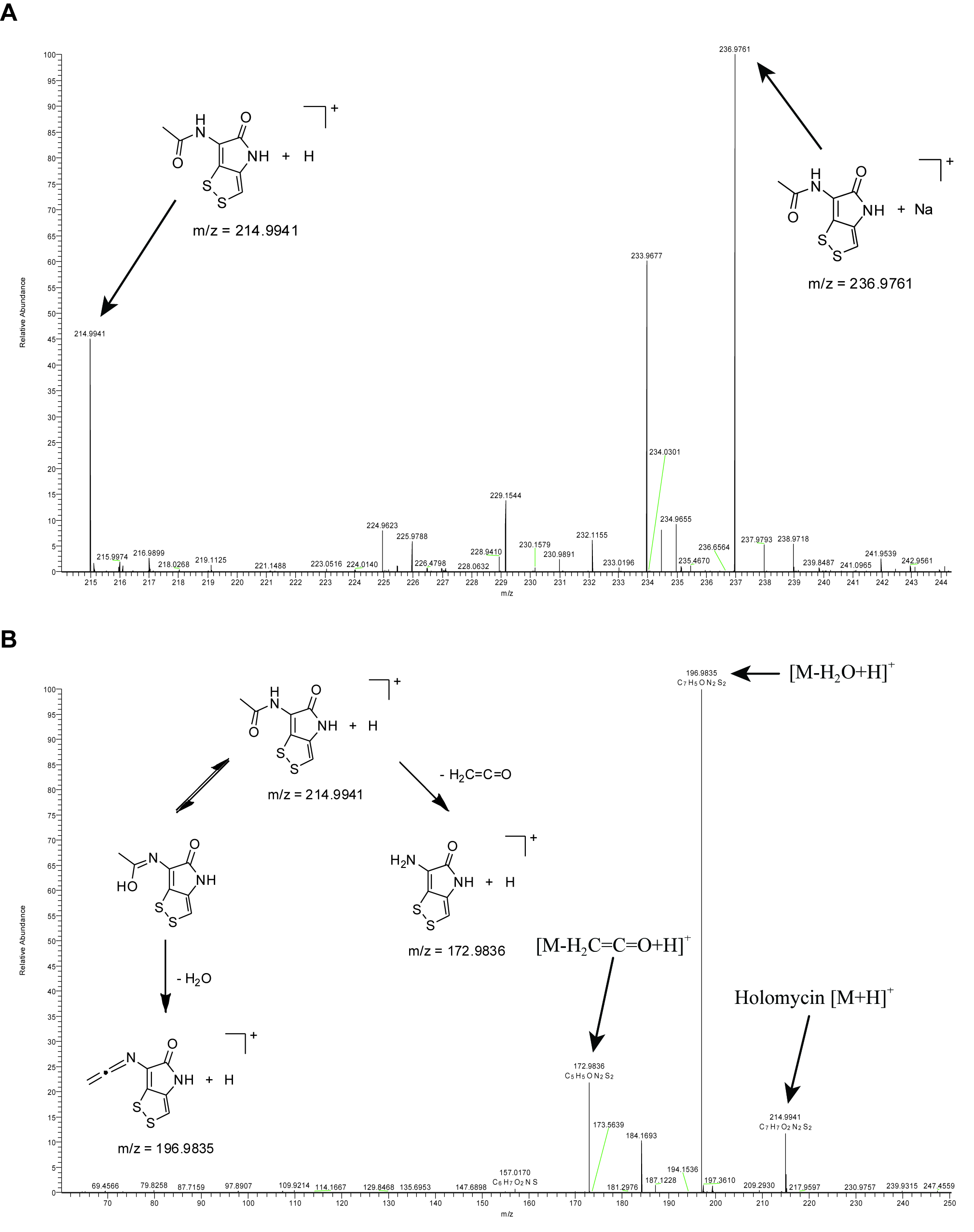

Supplement: Figure S3 — A. High-resolution ESI-MS of holomycin, the bioactive compound isolated from clavu7. [M+H]+, observed mass: 214.9941, calculated mass: 214.9943; [M+Na]+, observed mass: 236.9761, calculated mass: 236.9763. B. High-resolution ESI-MS/MS of holomycin (m/z 214.99) and key MS/MS fragments. There are three main peaks in the data: [M+H]+, observed mass: 214.9941, calculated mass: 214.9943; [M−H2O+H]+, observed mass: 196.9835, calculated mass: 196.9838; [M−COCH3+H]+, observed mass: 172.9836, calculated mass: 172.9838. A fragmentation mechanism leading to the latter ion, holothin, has been proposed [55]. (TIF) [file pone.0033727.s003.tif]

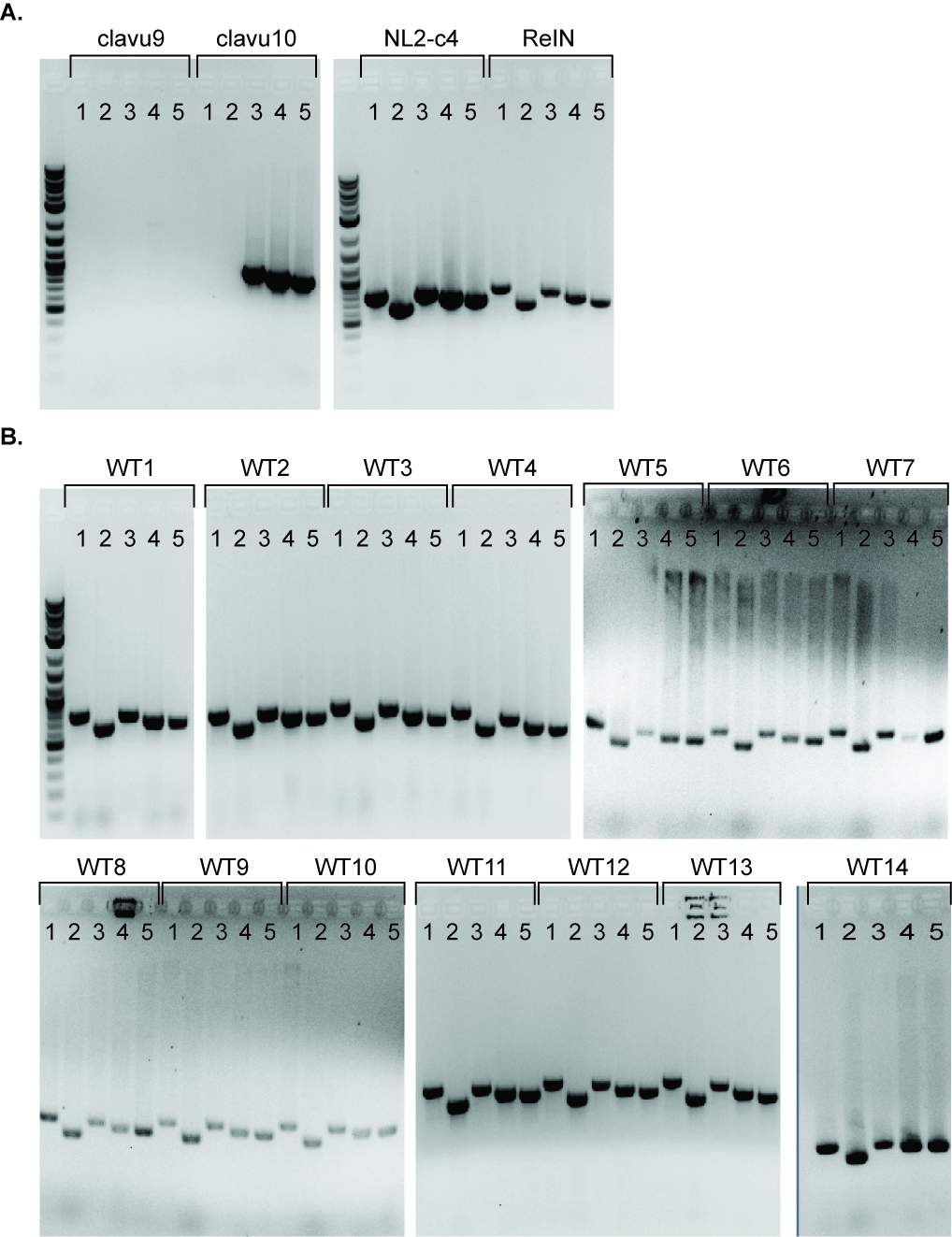

Supplement: Figure S4 — PCR amplification of five pSCL4 amplicons from additional strains evolved against MRSA N315 and from fourteen evolved control replicates. A. PCR amplification from evolved strains clavu9, clavu10, NL2-c4, and ReIN. Clavu9 does not contain the megaplasmid, and a portion of the 5′ end is missing from clavu10. Both NL2-c4 and ReIN contain the full length megaplasmid. B. PCR amplification from fourteen control replicates evolved in the absence of MRSA N315 over the same amount of time. All fourteen contain the megaplasmid. (TIF) [file pone.0033727.s004.tif]

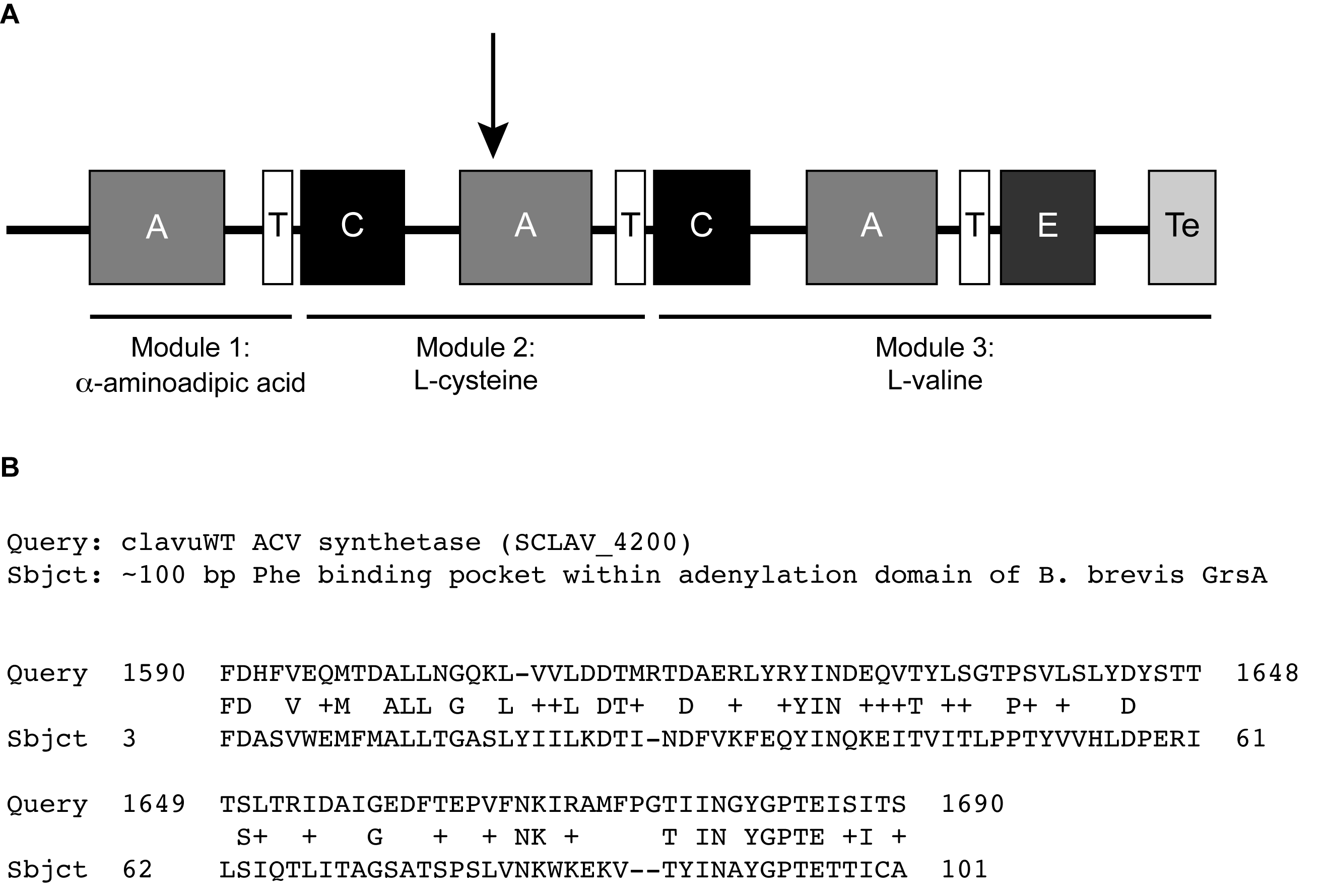

Supplement: Figure S5 — A. Module and domain architecture of S. clavuligerus ACV synthetase. There are three modules that activate each of the three amino acids. The first module contains two domains and initiates biosynthesis of the LLD-ACV tripeptide. The second and third modules each contain condensation (C) – adenylation (A) – thiolation (T) domains typically found in many non-ribosomal peptide synthetases. The final two domains in the third module are epimerization (E) and thioesterase (Te) domains. The arrow points to the location of the SNP detected in the clavu7 ACV synthetase. B. Alignment of the second adenylation domain of S. clavuligerus ACV synthetase with the amino acid binding pocket within the GrsA phenylalanine adenylation domain from B. brevis [73]. Based on the alignment, the l-cysteine binding pocket in ACV synthetase is predicted to be between residues 1590 and 1690. The putative V1525L substitution therefore lies within the second adenylation domain but to the N-terminal side of the predicted binding pocket [67], [68]. (TIF) [file pone.0033727.s005.tif]

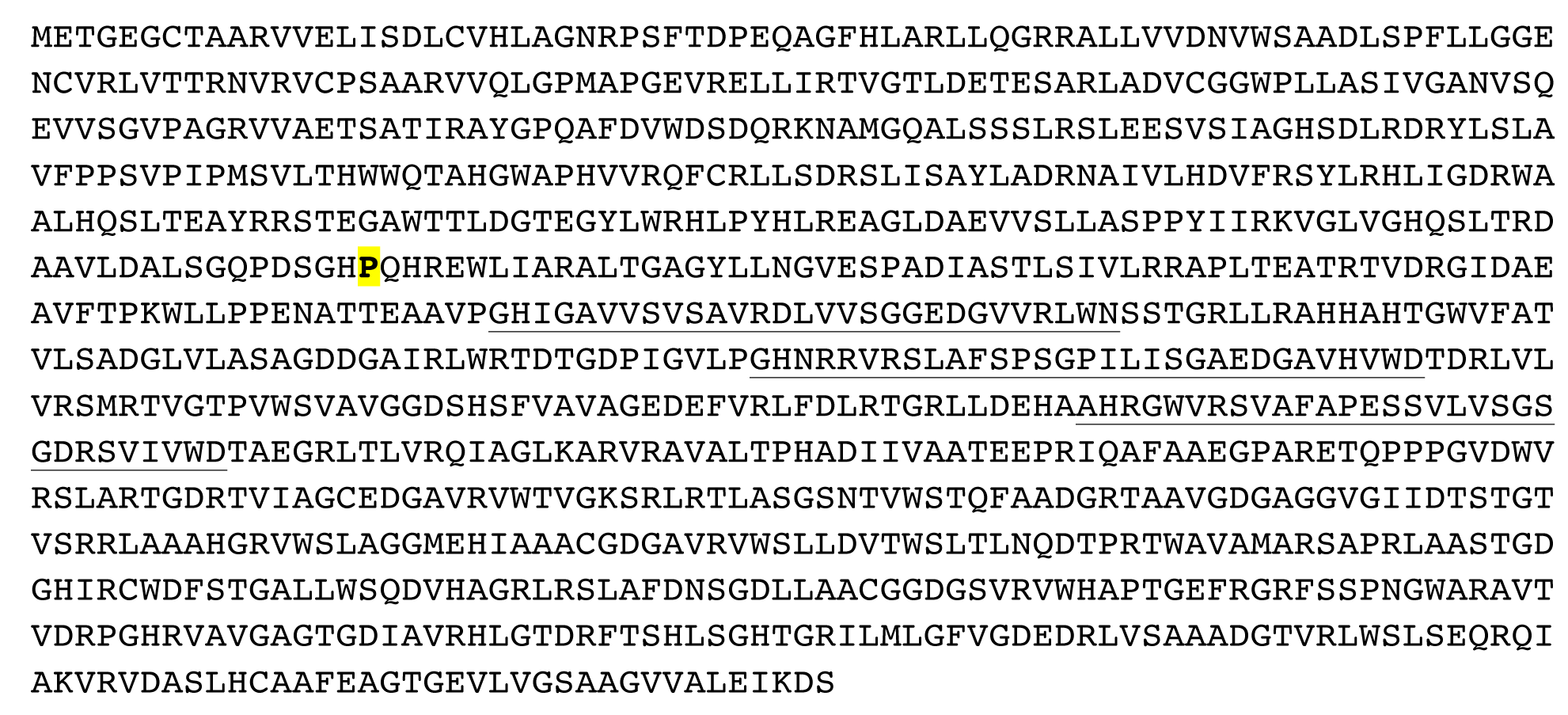

Supplement: Figure S6 — Location of the ∼27 bp conserved core unit (underlined) from three putative WD-40 repeats in SSCG_02612. The proline associated with the C1096T SNP detected in this gene in clavu7 (Table 1) is shown as well (highlighted); the SNP results in a P366S amino acid substitution. The annotation for SSCG_02612 is shorter than that for SCLAV_2674 (the latter contains an additional 182 amino acids at the N-terminus), but both have the same reading frame. (TIF) [file pone.0033727.s006.tif]
