# Supplementary material for: Exploiting Adaptive Laboratory Evolution of Streptomyces clavuligerus for Antibiotic Discovery and Overproduction
Source: PLoS One. 2012 Mar 21;7(3):e33727. doi: 10.1371/journal.pone.0033727 (PMC3312335; doi:10.1371/journal.pone.0033727)
Supplement: Table S1 — Primer sets used to PCR amplify five amplicons from pSCL4. (DOC) [file pone.0033727.s007.doc]

**Table S1**. Primer sets used to PCR amplify five amplicons from pSCL4.

|  | Locus Tag | Forward Primer | Reverse Primer |
| --- | --- | --- | --- |
| 1 | SCLAV_p0043 | CTGGTCCCTGACCCTCTACA | TGGCCTTGATGTTGAACAGA |
| 2 | SCLAV_p0395 | GAACTCCTGCTCGGTAGTGG | ATCGTGGGTGTACGAGAAGG |
| 3 | SCLAV_p0841 | GTCTGGTAGATGGGGAAGCA | TGATTGCGTCGATTCTCAAG |
| 4 | SCLAV_p1272 | GTGTGTCCGAGGTTGGACTT | AGTGATGCACGGCGACTAC |
| 5 | SCLAV_p1488 | ATTCGGGATACACGGTGAAC | TCGGAGACGAAGAAGGTGTT |

Locus IDs are from accession number ADGD01000000.
