# Supplementary material for: Exploiting Adaptive Laboratory Evolution of Streptomyces clavuligerus for Antibiotic Discovery and Overproduction
Source: PLoS One. 2012 Mar 21;7(3):e33727. doi: 10.1371/journal.pone.0033727 (PMC3312335; doi:10.1371/journal.pone.0033727)
Supplement: Table S2 — Primer sets used to confirm clavu7 SNPs by Sanger sequencing. (DOC) [file pone.0033727.s008.doc]

**Table S2**. Primer sets used to confirm clavu7 SNPs by Sanger sequencing.

|  | Locus Tag | Forward Primer | Reverse Primer |
| --- | --- | --- | --- |
| 1 | SSCG_02612 | CTGCTGTTCCACAACCTGAC | GAACCGAGGGCTACCTGTG |
| 2 | SSCG_05972 | GCCAGCTGTCTTACGGAGAT | GGAAGATGTCCGGGTACTGA |
| 3 | SSCG_00146 | GTGCCGGAGAGATAGGTGAC | TCGACAAGAACGAGCTGATG |
| 4 | SSCG_05988 | CTGGAGCTTCAGCGAGAAGT | CTGGAGTACACGGCTCCACT |
| 5 | SSCG_06722 | CGATGGTTTCTGGTCAACG | GGTGTTGTTGAGGTGGGTGT |
